# Plasma proteomic and metabolomic profiling of coronary and carotid atherosclerosis in the SCAPIS study—differences and similarities

**DOI:** 10.1093/cvr/cvaf076

**Published:** 2025-05-06

**Authors:** Tove Fall, Anders Gummesson, Ulf Hammar, Håkan Ahlström, Oskar Angerås, Anders Blomberg, John Brandberg, Kenneth Caidahl, Elin Chorell, Jan Edvin Engvall, Per Eriksson, David Erlinge, Bruna Gigante, Ola Hjelmgren, Johan Hultdin, Tomas Jernberg, Johan Kihlberg, Lars Lind, Martin Magnusson, Fredrik Hans Nyström, Carlo Pirazzi, Alexandru Schiopu, Johan Sundström, Stefan Söderberg, Carl Johan Östgren, Gunnar Engström

**Affiliations:** Department of Medical Sciences, Molecular Epidemiology, Uppsala University, Dag Hammarskiölds väg 14B, 752 37 Uppsala, Sweden; Department of Molecular and Clinical Medicine, Institute of Medicine, Sahlgrenska Academy, University of Gothenburg, Gothenburg, Sweden; Department of Clinical Genetics and Genomics, Region Västra Götaland, Sahlgrenska University Hospital, Gothenburg, Sweden; Department of Medical Sciences, Molecular Epidemiology, Uppsala University, Dag Hammarskiölds väg 14B, 752 37 Uppsala, Sweden; Science for Life Laboratory, Uppsala University, Uppsala, Sweden; Department of Surgical Sciences, Section of Radiology, Uppsala University, Uppsala, Sweden; Antaros Medical AB, BioVenture Hub, Mölndal, Sweden; Department of Molecular and Clinical Medicine, Institute of Medicine, Sahlgrenska Academy, University of Gothenburg, Gothenburg, Sweden; Department of Cardiology, Region Västra Götaland, Sahlgrenska University Hospital, Gothenburg, Sweden; Department of Public Health and Clinical Medicine, Umeå University, Umeå, Sweden; Department of Radiology, Institute of Clinical Sciences, Sahlgrenska Academy, University of Gothenburg, Gothenburg, Sweden; Department of Radiology, Region Västra Götaland, Sahlgrenska University Hospital, Gothenburg, Sweden; Department of Clinical Physiology, Karolinska University Hospital, and Karolinska Institute, Stockholm, Sweden; Department of Clinical Physiology, Sahlgrenska University Hospital, and Sahlgrenska Academy, Gothenburg, Sweden; Department of Public Health and Clinical Medicine, Umeå University, Umeå, Sweden; CMIV, Centre of Medical Image Science and Visualization, Linköping University, Linköping, Sweden; Department of Clinical Physiology, and Department of Health, Medicine and Caring Sciences, Linköping University, Linköping, Sweden; Division of Cardiovascular Medicine, Department of Medicine Solna, Karolinska Institute, Stockholm, Sweden; Karolinska University Hospital Solna, Stockholm, Sweden; Department of Clinical Sciences Lund, Cardiology, Lund University, and Skåne University Hospital, Lund, Sweden; Division of Cardiovascular Medicine, Department of Medicine, Karolinska Institute, Stockholm, Sweden; Department of Cardiology, Danderyd Hospital, Stockholm, Sweden; Department of Molecular and Clinical Medicine, Institute of Medicine, Sahlgrenska Academy, University of Gothenburg, Gothenburg, Sweden; Pediatric Heart Centre, Queen Silvia Children's Hospital, Sahlgrenska University Hospital, Region Västra Götaland, Gothenburg, Sweden; Department of Medical Biosciences, Clinical Chemistry, Umeå University, Umeå, Sweden; Department of Clinical Sciences, Danderyd University Hospital, Karolinska Institute, Stockholm, Sweden; CMIV, Centre of Medical Image Science and Visualization, Linköping University, Linköping, Sweden; Department of Radiology and Department of Health, Medicine and Caring Sciences, Linköping University, Linköping, Sweden; Department of Medical Sciences, Clinical Epidemiology, Uppsala University, Uppsala, Sweden; Department of Clinical Sciences, Lund University, Malmö, Sweden; Department of Cardiology, Skåne University Hospital, Malmö, Sweden; Wallenberg Center for Molecular Medicine, Lund University, Lund, Sweden; Faculty of Health Sciences, North-West University, Hypertension in Africa Research Team (HART), Potchefstroom, South Africa; Department of Health, Medicine and Caring Sciences, Linköping University, Linköping, Sweden; Department of Cardiology, Region Västra Götaland, Sahlgrenska University Hospital, Gothenburg, Sweden; Department of Clinical Sciences, Lund University, Malmö, Sweden; Department of Internal Medicine, Skåne University Hospital, Lund, Sweden; Department of Medical Sciences, Uppsala University, Uppsala, Sweden; The George Institute for Global Health, University of New South Wales, Sydney, Australia; Department of Public Health and Clinical Medicine, Umeå University, Umeå, Sweden; CMIV, Centre of Medical Image Science and Visualization, Linköping University, Linköping, Sweden; Department of Health, Medicine and Caring Sciences, Linköping University, Linköping, Sweden; Department of Clinical Sciences, Lund University, Malmö, Sweden

**Keywords:** Proteomics, Metabolomics, Atherosclerosis

**Time of primary review: 44 days**

The identification of circulating biomarkers of atherosclerotic disease in coronary and carotid vascular beds can provide insights into molecular mechanisms and facilitate early diagnosis and risk stratification. It has been suggested that proteomics and metabolomics could moderately enhance the prediction of cardiovascular events beyond traditional risk factors, but few markers are robustly replicated.^1–3^ Moreover, the combined ability of proteomics and metabolomics to classify atherosclerosis and any potential differences across vascular beds status is less understood. The aim of this study was to identify circulating -omics biomarkers of image-based measures of coronary and carotid atherosclerosis to point to potential mechanistic pathways and to assess whether these biomarkers can aid in the classification of an individual as having atherosclerosis or not.

This cross-sectional study included a subset of 4943 individuals aged 50–65 from the population-based Swedish CArdioPulmonary bioImage Study (SCAPIS) whose plasma samples had undergone targeted proteomics and nuclear magnetic resonance-(NMR) based metabolomics analysis and who had completed coronary computed tomography angiography and ultrasound examinations of the carotid arteries.^4^ Validation of results was conducted in data from 1026 participants from the SCAPIS-Pilot study from the Gothenburg site.^5^ Plasma samples were analyzed at the Clinical Biomarkers Facility, Science for Life Laboratory, Uppsala University, with the proximity extension assay (PEA) technique using the Olink Multiplex CVD II and III 96 × 96 panel, two high-specificity assays that simultaneously measure concentrations of 92 cardiovascular candidate proteins each.^6^ We excluded 19 proteins with measurements missing, had a warning in the laboratory quality control, or had values below LOD in >15% of samples. Values < LOD in remaining proteins were kept. Proteins were then normalized by plate on a log_2_-scale at the laboratory. Plasma was also sent for measurements of 225 metabolic parameters including concentrations, lipoprotein particle size and metabolite ratios with nuclear magnetic resonance spectroscopy (NMR) at Nightingale Health, Finland.^7^ All metabolites had <15% values below LOD. We included the 146 measurements of metabolite concentrations and lipoprotein particle size and excluded metabolite ratios. Metabolites that were measured as concentrations (k = 142) were log_2_-transformed before all analysis. If a metabolite had a measured value of 0, we imputed this value using half of the minimum non-zero value in the dataset before transformation. This imputation was done in main and pilot data separately.

First, we assessed the association of each protein/metabolite with coronary and carotid atherosclerosis in a series of proportional odds ordinal regression models using three sets of covariates. In the primary analysis, coronary atherosclerosis was defined as coronary artery calcium score (CACS) and carotid atherosclerosis as absent, unilateral, or bilateral^5^ plaques (carotid intima media thickness ≥1.5 mm) from 2-dimensional greyscale ultrasound images. The basic model was adjusted for age, sex, visit date and study centre; the extended model for basic model covariates and body mass index, diabetes, fasting glucose, smoking, systolic blood pressure (SBP), diastolic blood pressure (DBP), kidney function, medication for hypertension; and the lipid model for extended model covariates and non-HDL cholesterol and medication for dyslipidemia. We tested whether the coefficients for the carotid and coronary extended models were different by bootstrapping.

Secondly, we developed two sets of classification models with coronary and carotid atherosclerosis as a binary outcome (no atherosclerosis vs. atherosclerosis) applying Lasso regression. We built a logistic regression model including only established cardiovascular risk factors and a set of models including cardiovascular risk factors, metabolites, and proteins. Risk factors included age, total cholesterol, HDL cholesterol, SBP, hypertension medication, smoking and diabetes, study centre, interactions between sex and all other variables, between hypertension medication and SBP and a three-way interaction between hypertension medication, SBP, and sex. The performance of the models was evaluated by nested 10-fold cross-validation and compared using the likelihood ratio test for data with multiple imputations.^8^

The considered proteins had similar overall associations with coronary and carotid atherosclerosis. For coronary atherosclerosis, we found associations with eleven previously reported cardiovascular-associated proteins related to the renin-angiotensin system (renin and angiotensin-converting enzyme 2), tissue remodelling (matrix metalloproteinase-12 and osteoprotegerin), cholesterol metabolism (proprotein convertase subtilisin/kexin type 9), coagulation (tissue factor pathway inhibitor), inflammation and apoptosis (growth/differentiation factor 15), cell adhesion, migration, and immune response modulation (Galectin-4, stem cell factor, C-C motif chemokine 16) and soluble receptors for advanced glycosylation end products (*Figure 1*). Stem cell factor, tissue factor pathway inhibitor, and osteoprotegerin were associated with atherosclerosis in both vascular beds in the extended model, supporting shared pathophysiological pathways across vascular beds (*Figure 1*). We found 63 proteins associated with carotid atherosclerosis in the basic model, 17 in the extended model and 10 in the lipid-adjusted model. Of these 17, 4, and 0 replicated, respectively. Sensitivity analysis using non-imputed metabolite values rendered similar results with Spearman correlations between the odds ratios for the imputed metabolites and the odds ratios for the non-imputed metabolites being ≥0.98 for both carotid plaque and CACS in the extended model with slightly smaller effect sizes in the imputed model.

**Figure 1.**
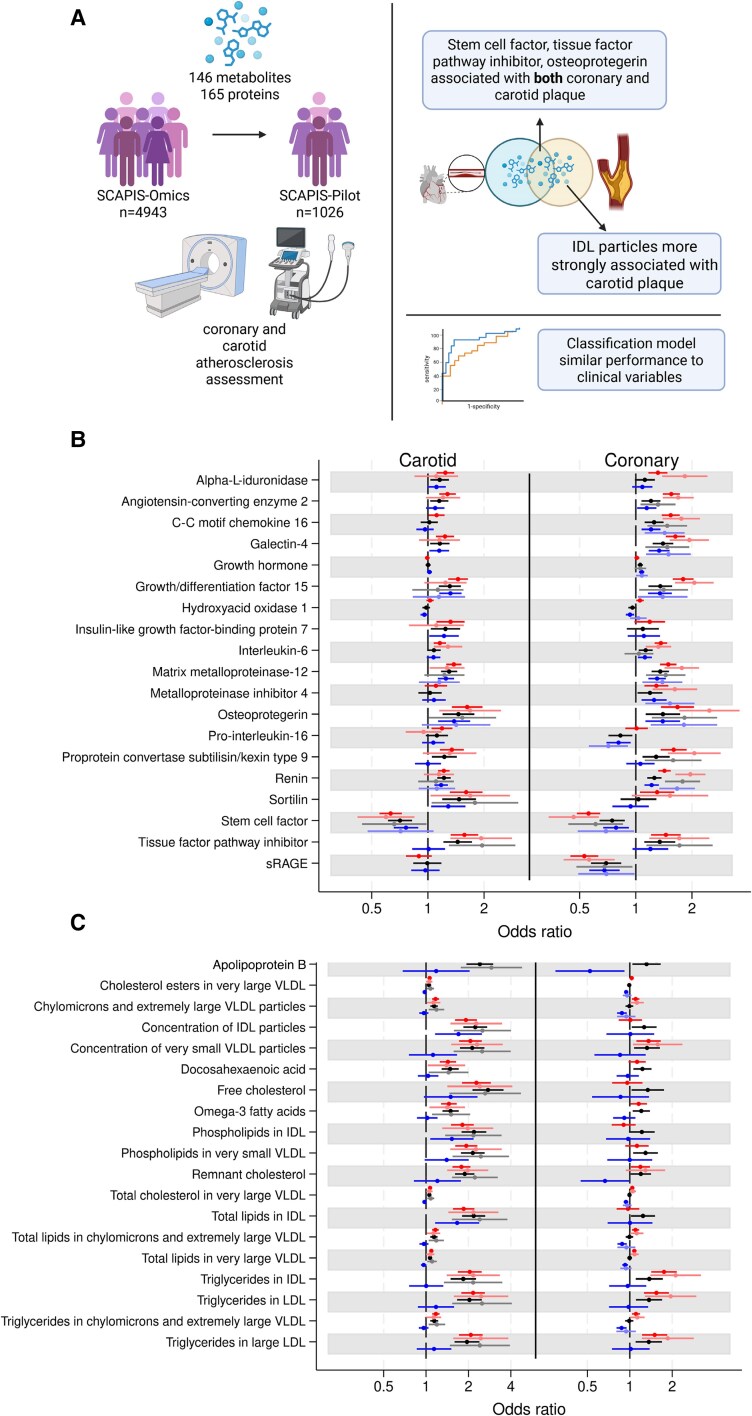
(*A*) Graphical abstract of the study with main results, Created in BioRender. Fall, T. (2025) https://BioRender.com/c83b659 (*B*) association of proteins and (*C*) metabolites with coronary and carotid atherosclerosis in up to 4925 individuals. The basic model (red) was adjusted for age, sex, visit date and study centre; the extended model (black) for basic model covariates and BMI, diabetes, fasting glucose, smoking, SBP, DBP, kidney function, medication for hypertension; and the lipid model (blue) for extended model covariates and non-HDL cholesterol and medication for dyslipidaemia. Lighter colours are from the replication study in SCAPIS-Pilot in up to 1026 individuals. Error bars represent 95% confidence intervals. The plot includes the 19 proteins and metabolites with lowest *P*-values in either the extended or lipid model for either coronary or carotid atherosclerosis and at least one replicated model.

The associations of metabolite parameters with atherosclerosis in the two vascular beds were different for 37 highly correlated metabolites, such as for intermediate-density lipoprotein particles (IDL), where the OR for coronary was 1.26 (95% CI 1.03–1.55) and for carotid 2.24 (95% CI 1.84–2.72) in the extended covariate model. Total lipids in IDL, concentration of IDL particles, Apolipoprotein B, and remnant cholesterol were strongly related to carotid atherosclerosis. Two alternative outcomes for coronary atherosclerosis were assessed in the extended model, the segment involvement score (SIS), and obstructive coronary atherosclerosis (at least one vessel with >50% obstruction, prevalence 6.0%). Beta coefficients for SIS were highly correlated to those for CACS, 0.96 for proteins, and 0.93 for metabolites. The corresponding correlations for obstructive coronary atherosclerosis were 0.65 and 0.67, respectively.

The likelihood ratio test showed a significant gain in adding proteins and metabolites to the model built on cardiovascular risk factors for the carotid (*P* < 0.001) and the coronary model (*P* = 0.006) in the discovery cohort. However, when applying the model to SCAPIS-Pilot, the discrimination performance [C-statistics coronary 0.76 (95% CI: 0.73–0.79); carotid 0.63 (0.59–0.66)] was similar to the model including cardiovascular risk factors only [C-statistics coronary 0.76 (95% CI: 0.73–0.79); carotid 0.62 (0.59–0.66)]. These results are in line with a recent study on image-based atherosclerosis, where another smaller subset of SCAPIS (*n* = 883) with a larger protein panel available acted as the replication study.^9^ Strengths compared to that and earlier similar studies includes a large training dataset, a model based on two types of omics data and the inclusion of data on atherosclerosis in two vascular beds. A limitation of the present study was that only two protein panels were available. On a similar note, metabolite profiling with NMR only captures abundant metabolites, whereas a mass-spectrometry-based method would capture also more rare metabolites.

In conclusion, we identified associations of 11 plasma proteins related to key processes in cardiovascular disease such as renin-angiotensin system, tissue remodelling, cholesterol metabolism, coagulation and inflammation to be robustly associated with coronary atherosclerosis after adjustments for lifestyle, non-HDL cholesterol, and multiple comparisons. These findings expand previous studies that largely focused on clinical events rather than image-based measures in a largely healthy population. Moreover, we observed associations of a large number of highly correlated metabolites primarily related to IDL and LDL cholesterol with carotid atherosclerosis. However, these were attenuated and non-significant after adjustments for non-HDL cholesterol and lipid-lowering treatment, indicating that the detailed NMR measurement of these do not add more information than a standard measurement of non-HDL-cholesterol.

The results indicate similar proteomic profiles for atherosclerosis in carotid and coronary vessels, while the relationships with plasma lipids differed between the vascular beds. The differences in association of plasma lipids with carotid atherosclerosis has unclear clinical implications as studies on risk of incident events has shown larger effect estimates for myocardial infarction than for stroke for the lipids implicated in the current study.^10^ The measured proteins and metabolites did not improve the classification of atherosclerosis status but should be explored as potential pathways. While our study focused on established measures of atherosclerosis like CACS, SIS, and stenosis degree, future research should incorporate an assessment of adverse plaque features in non-obstructive lesions, as these characteristics have been shown to significantly impact clinical outcomes and may provide additional prognostic value beyond traditional atherosclerosis metrics.^11^

## Data Availability

Access to pseudonymized SCAPIS phenotype data requires ethical approval from the Swedish Ethical Review Board and approval from the SCAPIS Data access board (https://www.scapis.org/data-access/). All participants provided written informed consent. The study was conducted following the Declaration of Helsinki. The Swedish Ethical Review Authority approved the Swedish CardioPulmonary bioImage Study (DNR 2010-228-31 M) and (DNR 2017/183-31).
